# Small molecule nAS‐E targeting cAMP response element binding protein (CREB) and CREB‐binding protein interaction inhibits breast cancer bone metastasis

**DOI:** 10.1111/jcmm.14024

**Published:** 2018-11-20

**Authors:** Min Jiang, Yufei Yan, Kai Yang, Zhuochao Liu, Jin Qi, Hanbing Zhou, Niandong Qian, Qi Zhou, Tianqi Wang, Xing Xu, Xiangshu Xiao, Lianfu Deng

**Affiliations:** ^1^ Shanghai Key Laboratory for Bone and Joint Diseases Shanghai Institute of Traumatology and Orthopaedics, Shanghai Ruijin Hospital, Shanghai Jiaotong University School of Medicine Shanghai China; ^2^ Program in Chemical Biology, Department of Physiology and Pharmacology Oregon Health & Science University Portland Oregon

**Keywords:** breast cancer bone metastasis, CBP, CREB, naphthol AS‐E, osteoclasts

## Abstract

Bone is the most common metastatic site for breast cancer. The excessive osteoclast activity in the metastatic bone lesions often produces osteolysis. The cyclic‐AMP (cAMP)‐response element binding protein (CREB) serves a variety of biological functions including the transformation and immortalization of breast cancer cells. In addition, evidence has shown that CREB plays a key role in osteoclastgenesis and bone resorption. Small organic molecules with good pharmacokinetic properties and specificity, targeting CREB‐CBP (CREB‐binding protein) interaction to inhibit CREB‐mediated gene transcription have attracted more considerations as cancer therapeutics. We recently identified naphthol AS‐E (nAS‐E) as a cell‐permeable inhibitor of CREB‐mediated gene transcription through inhibiting CREB‐CBP interaction. In this study, we tested the effect of nAS‐E on breast cancer cell proliferation, survival, migration as well as osteoclast formation and bone resorption in vitro for the first time. Our results demonstrated that nAS‐E inhibited breast cancer cell proliferation, migration, survival and suppressed osteoclast differentiation as well as bone resorption through inhibiting CREB‐CBP interaction. In addition, the in vivo effect of nAS‐E in protecting against breast cancer‐induced osteolysis was evaluated. Our results indicated that nAS‐E could reverse bone loss induced by MDA‐MB‐231 tumour. These results suggest that small molecules targeting CREB‐CBP interaction to inhibit CREB‐mediated gene transcription might be a potential approach for the treatment of breast cancer bone metastasis.

## 1 INTRODUCTION

Breast cancer is the most commonly diagnosed cancer among females.^1^ At least 80% of the breast cancer patients will develop bone metastases during the course of their diseases.^2^, ^3^ In the metastatic bone lesions, there are complex cross‐talks between metastatic breast cancer cells and bone cells.^4^, ^5^, ^6^, ^7^ For example, breast cancer cells produce various secreted factors^8^, ^9^, ^10^, ^11^ in the bone microenvironment, which enhance osteoclastogenesis and inhibit osteoblastogenesis, Patients who develop bone metastases suffer from skeletal‐related events (SREs)^12^ such as pathological fracture, spinal cord compression, bone pain and hypercalcemia. Because breast cancer bone metastasis has a generally osteolytic nature,^13^, ^14^ inhibitors of osteoclastic bone resorption including bisphosphonates and denosumab have become the most commonly used medications in the treatment of breast cancer‐induced osteolysis.^15^, ^16^, ^17^ However, their effects are relatively moderate in clinical studies.^18^, ^19^ It is also reported that bisphosphonates have serious adverse effects such as osteonecrosis of the jaw and subtrochanteric fractures.^20^ Denosumab, a monoclonal antibody against receptor activator of NF‐kB ligand (RANKL), has been shown to increase risk of pancreatitis and serious infections including endocarditis, erysipelas and infectious arthritis.^21^ Therefore, novel agents have been pursued to mitigate osteolysis induced by breast cancer metastasis. Cathepsin K inhibitor odanacatib has been reported to be an agent against osteoporosis and breast cancer‐induced bone metastasis.^22^, ^23^ However, odanacatib was withdrawn from the clinical trials for safety reasons. In addition, clinical studies of c‐Src inhibitors have been initiated in patients with bone metastases, since c‐Src plays multiple roles in the bone resorption and in the proliferation, survival, metastasis of breast cancer cells.^24^, ^25^ Unfortunately, the efficiency of c‐Src inhibitors has not been generally encouraging in clinical trials to date. Hence, other safe and efficient targets and new strategies in the treatment of breast cancer bone metastasis are needed.

The cyclic‐AMP (cAMP)‐response element binding protein (CREB) is a nuclear transcription factor belonging to a family of basic leucine zipper (bZIP)‐containing transcription factors that serves a variety of biological functions including cellular proliferation and differentiation.^26^, ^27^, ^28^ Recently, accumulating evidence has revealed that CREB participates in the immortalization and transformation of cancer cells. It is also reported that CREB and phosphorylated CREB (p‐CREB) have been shown to be consistently over‐expressed in breast cancer tissues.^29^, ^30^, ^31^, ^32^ CREB regulates a number of critical genes involved in cellular proliferation, anti‐apoptosis and metastasis of breast cancer.^33^, ^34^ These include b‐cell lymphoma 2 (Bcl‐2), vascular endothelial growth factor and type IV collagenase matrix metalloproteinase 2. On the other hand, evidence has also shown that CREB plays a key role in the differentiation of osteoclast and bone resorption.^35^, ^36^ CREB activation is required for the inductions of *c*‐Fos and nuclear factor of activated T‐cells cytoplasmic 1 (NFATc1), which control the expressions of bone resorption enzymes such as tartrate‐resistant acid phosphatase (TRAP), matrix metalloprotein‐9 and cathepsin K. In the early‐phase of osteoclast differentiation, the expression of *c*‐Fos is induced after CREB activated is phosphorylated and activated by Calcium/calmodulin‐dependent protein kinase IV, which is induced by Ca^2+^ signalling. In the late‐phase of osteoclast differentiation, NFATc1 is dephosphorylated by calcineurin which is also induced by Ca^2+^ signalling pathway, translocates into the nucleus and interacts with p‐CREB to activate the transcription of osteoclast‐specific genes.^37^ When A‐CREB, a dominant‐negative form of CREB, was introduced into osteoclast precursor cells, the formation of TRAP‐positive osteoclasts and the expression of NFATc1 were significantly reduced.^37^

Drugs serving as “dual inhibitors” for the prevention of breast cancer‐induced bone metastasis and osteolysis are expected to improve therapeutic efficacy. Considering the key roles of CREB in breast cancer and osteoclastogenesis, we proposed inhibition of CREB activity as an intriguing strategy for the development of novel breast cancer bone metastases therapeutics. Biological approaches were pursued to inhibit CREB function in breast cancer cells such as the utilization of dominant‐negative CREB mutants, CREB “decoy” oligonucleotides and RNA interference.^38^, ^39^, ^40^, ^41^ However, clinical applications of these approaches are rather limited since gene therapy techniques would also be required.^42^ Therefore, utilizing small organic molecules to prevent breast cancer‐induced bone metastasis and osteolysis could be of great interest due to their better pharmacokinetic properties. There are three potential intervention points for small molecules as chemical inhibitors of CREB‐mediated gene transcription. The first approach, targeting CREB‐related kinases involves in the use of kinase inhibitors to inhibit CREB phosphorylation and its transcription. Unfortunately, this approach elicits many off‐target effects.^43^ The second one is to inhibit CREB‐CRE interaction. It is reported that NSC 12155 and NSC 45576 were identified as inhibitors of CREB‐CRE interaction by high‐throughput screening assay from the NCI‐diversity set of 1900 compounds. However, these compounds are not specific in inhibiting CREB‐CRE interaction.^44^ The third strategy is to target CREB‐binding protein (CREB‐CBP) interaction to inhibit CREB‐mediated gene transcription in breast cancer cells and osteoclasts.

CREB is not activated until it is phosphorylated at Ser133 and its subsequent binding to CBP through kinase‐inducible domain (KID) in CREB and KID‐interacting (KIX) domain in CBP. The binding interface between the KID‐KIX interaction is structurally well‐characterized by NMR spectroscopy.^45^ In our previous study, we described naphthol AS‐E (nAS‐E) as a cell‐permeable small molecule inhibitors of KIX‐KID interaction to inhibit CREB‐mediated gene transcription based on a renilla luciferase complementation assay in HEK293T cells.^46^, ^47^ In this study, we further verified the binding modes of nAS‐E in KIX‐KID interface by molecular docking and investigated the inhibition of nAS‐E on CREB‐CBP interaction in osteoclasts. We also studied its inhibition effects on the proliferation, migration and apoptosis on MDA‐MB‐231 breast cancer cells and on the osteoclast formation, bone resorption as well. Thus, our results highlighted the potential of small molecules targeting CREB‐CBP interaction in the treatment of breast cancer‐induced bone loss.

## 2 MATERIALS AND METHODS

### 2.1 Materials

All reagents used were of analytical grade and obtained commercially. Naphthol AS‐E was purchased from Aladdin. Dimethyl sulfoxide (DMSO) and TRAP staining kit were purchased from Sigma. Cell culture reagents such as α‐modified eagle’s medium (α‐MEM), DMEM, foetal bovine serum (FBS), penicillin and streptomycin (PS) were from Gibco. Cytokines macrophage‐colony stimulating factor (M‐CSF) and RANKL were obtained from Peprotech. TRIZOL was from Invitrogen, reverse transcript reagents were from TAKARA. SYBR Green PCR Master Mix was from Novoprotein. TRAP activity assay kit was from Beyotime Biotechnology. Bone slices were from bovine femur and cut into 100 μm thick. Antibodies for p‐CREB, CREB, CBP, *c*‐fos, NFATc1 and actin were from CST. Transwell chambers were obtained from Millipore. Kit for flow cytometry was from Invitrogen.

### 2.2 Cell culture

MDA‐MB‐231 cell line was purchased from ATCC. All cells were cultured at 37°C in a 5%‐humidified CO_2_ incubator. Bone marrow monocytes (BMMs) isolated from C57/BL6 mouse were cultured in α‐MEM containing 10% FBS, 100 U/mL penicillin and 100 g/mL streptomycin (full α‐MEM), MDA‐MB‐231 cell line was cultured in DMEM (4.5 g/mL glucose) containing 10% FBS, 100 U/mL penicillin and 100 g/mL streptomycin (full DMEM).

### 2.3 Isolation of BMMs

Bone marrow monocytes for osteoclast differentiation were isolated from 4‐week‐old male C57/BL6 mouse as previously described.^48^ Briefly, all the bone marrow cells in the femur and tibiae of mouse were flushed out by α‐MEM and then incubated in full α‐MEM overnight. The cells in the supernatant were collected and then cultured in full α‐MEM containing 30 ng/mL M‐CSF for proliferation. After reaching 90% confluence, cells adhering to the bottom of the dish classified as BMMs were harvested by cell scraper. BMMs were induced to osteoclasts by full α‐MEM plus M‐CSF (30 ng/mL) and RANKL (50 ng/mL).

### 2.4 Animal

C57BL/6 mice (4‐week old; male) were purchased from SLAC laboratory and BALB/cA nude mice (5‐6 weeks old; female) were purchased from Shanghai Sippr‐BK Laboratory Animal Co. Ltd. Animals were maintained at 22‐24°C and 55%‐60% humidity in a room with a 12/12‐h light/dark cycle. All animal experiments were conducted according to the guidelines of the humane use and care of laboratory animals and were approved by the Shanghai Jiao Tong University School of Medicine Animal Study Committee.

### 2.5 Molecular modelling

Molecular docking was operated with Autoock Vina with default settings. The protein structure was prepared using Chimera (https://www.rbvi.ucsf.edu/chimera)^49^ and molecular docking was performed with Autoock Vina with default settings.^50^ The docking results were viewed using Pymol0.99rc6. The NMR structures of CREB‐CBP complex are downloaded from www.RCSB.org (PDB code: 1KDX). Chain A is KIX domain of CBP and chain B is KID. By analysing the interaction interface of KIX‐KID, a relatively deep pocket was found on the surface of KIX structure (state 8 of NMR structures) close to residues ILE137 and Leu141 of KID.

### 2.6 Western blotting

Cells were treated as indicated, then washed and lysed. The lysates were subsequently subjected to SDS‐PAGE. Immunoblot analyses were performed with primary antibodies purchased from CST. The secondary antibodies were visualized using an Immobilon Western kit (Millipore).

### 2.7 Co‐immunoprecipitation

Immunoprecipitation (IP) buffer (1% Triton X‐100, 150 mmol/L NaCl, 10 mmol/L Tris, pH 7.4, 1 mmol/L EDTA, 1 mmol/L EGTA, pH 8.0, 0.2 mmol/L sodium ortho‐vanadate, 1 mmol/L PMSF, 0.5% protease inhibitor cocktail, 0.5% IGEPAL CA‐630) were used to lysed BMMs. The cell lysates were then centrifuged at 16 000 *g* for 30 minutes at 4°C and the supernatants were incubated with p‐CREB antibodies overnight, followed by incubation with Protein A/G‐coated agarose beads (Merck) for another 4 hours at 4°C. Then samples were washed with cold IP buffer three times and the supernatants were removed by centrifugation at 2000 *g* for 1 minute. The proteins were then separated from the beads using IB loading buffer for 5 minutes at 95°C. The supernatants were collected and were detected with indicated antibodies by blotting and re‐blotting.

### 2.8 MTT assay

The proliferation effects of nAS‐E on different cells were determined by MTT assay. Cells were plated to 40%‐50% confluence per well in 96‐well plates in full medium and cultured overnight. Then, the cells were treated with indicated concentrations of nAS‐E in triplicate for indicated hours. After that, 100 μL MTT was added to each well and the plates were incubated at 37°C for 2 hours. Then, the supernatant was removed and the crystals were dissolved in 100 μL DMSO. Optical density (OD) was measured with an Infinite F200 PRO absorbance microplate reader (Tecan) at 570 nm. Cell viability was calculated relative to the control.

### 2.9 Cell migration assay

To assess cell migration potential, 5 × 10^4^ cells in 100 μL of serum‐free medium were plated in the upper chamber of transwell migration chambers (0.8 μm pore; Millipore). The lower chamber was filled with 500 μL of serum‐free medium with the indicated nAS‐E. After 24 hours, the upper chamber was removed and cells on the lower surface of the filter were fixed with methanol and stained with crystal violet solution. The images of stained cells were captured under a microscope. Then the crystal violet was dissolved in 100 μL of 33% acetic acid. OD was measured with an Infinite F200 PRO absorbance microplate reader (Tecan) at 570 nm. Data were expressed relative to the control.

### 2.10 Apoptosis assay

The apoptosis effect of nAS‐E on MDA‐MB‐231 cells was determined with the Apoptosis Assay Kit (Invitrogen, USA). Cells were treated with indicated concentrations of nAS‐E for 2 days. Then cells were washed twice with cold PBS and pelleted; the supernatants were discarded and the cells were resuspended in annexin binding buffer. Early apoptosis was detected by staining with Alexa Fluor 488 annexin V and propidium iodide. FACS was performed with a FACS can flow cytometer (Becton‐Dickinson, USA) and data were analysed with FlowJo software.

### 2.11 Tartrate‐resistant acid phosphatase colorimetric assay

Bone marrow monocytes were seeded in 96‐well plates at a density of 3 × 10^3^ cells/well. Cells were exposed to in full medium plus 30 ng/mL M‐CSF and 50 ng/mL RANKL in combinations with indicated concentrations of compounds in triplicate for 2 days. Cells were lysed and TRAP activity was measured by TRAP activity assay kit according to the manufacturer’s instructions. Briefly, the cell culture media were removed and cells were washed by PBS for three times. Then, cells were lysed by passive lysis buffer (Promega) for 15 minutes at 37°C and the supernatant were collected and incubated with para‐nitrophenylphosphate (p‐NPP) in the presence of disodium tartrate for 45 minutes. The reaction was subsequently stopped with the addition of NaOH. The TRAP activity was then quantified by measuring OD at 405 nm with Tecan absorbance microplate reader.

### 2.12 In vitro osteoclastogenesis assay

Bone marrow monocytes were cultured in 96‐well plates in full medium containing M‐CSF and allowed to adhere overnight. The medium was replaced and the cells were treated with 30 ng/mL M‐CSF and 50 ng/mL RANKL for additional 5 days. The medium was replaced every 2 days. TRAP staining was then performed with a leucocyte acid phosphatase kit (Sigma) according to the manufacturer’s instructions. TRAP‐positive multinucleated cells (≥3 nuclei) were scored as osteoclasts.

### 2.13 Bone resorption assay

For the bone resorption assay, BMMs were seeded on bovine femur bone slices in 96‐well plates and the next day cells were induced with 30 ng/mL M‐CSF and 50 ng/mL RANKL in combination with indicated concentrations of compound for 5 days. The medium was replaced for each 2 days. Cells were then fixed with 2.5% glutaraldehyde. Bone slices were stained by 0.5% toluidine blue and then imaged using an Olympus microscope with 200× magnification. Three fields were randomly selected for each bone slice for further pit area analyses which were quantified using Image J software.

### 2.14 RNA isolation and quantitative real‐time PCR

Total RNA extraction and mRNA expression analysis by quantitative real‐time (qRT)‐PCR were performed as described previously. mRNA levels of TRAP, V‐ATP, NFATc1, cathepsin K and β‐actin were quantified by qRT‐PCR using specific primers. The primer sequences were summarized below (Table 1). All values were reported as mean ± SD of triple measurements of each cDNA sample. mRNA levels were normalized to β‐actin mRNA.

**Table 1:** Sequences of all primers in quantitative RT‐PCR analysis

Gene	Primer sequence
NFATc1	Forward 5’‐ACCACCTTTCCGCAACCA‐3’ Reverse 5’‐TTCCGTTTCCCGTTGCA‐3’
V‐ATPase‐d2	Forward 5’‐ATGCTTGAGACTGCAGAG‐3’ Reverse 5’‐TTATAAAATTGGAATGTAGCT‐3’
TRAP	Forward 5’‐TCCCCAATGCCCCATTC‐3’ Reverse 5’‐CGGTTCTGGCGATCTCTTTG‐3’
MMP‐9	Forward 5’‐CAAACCCTGCGTATTTCC‐3’ Reverse 5’‐AGAGTACTGCTTGCCCAGGA‐3’
CtsK	Forward 5’‐GAAGAAGACTCACCAGAAGCAG‐3’ Reverse 5’‐TCCAGGTTATGGGCAGAGATT‐3’
β actin	Forward 5’‐CTGTCCCTGTATGCCTCTG‐3’ Reverse 5’‐ATGTCACGCACGATTTCC‐3’

NFATc1, nuclear factor of activated T‐cells cytoplasmic 1; TRAP, tartrate‐resistant acid phosphatase; MMP‐9, matrix metalloprotein‐9.

### 2.15 MDA‐MB‐231 and BMMs co‐culture assay

Bone marrow monocytes were seeded at 5 × 10^3^ cells/well with 30 ng/mL M‐CSF and allowed to adhere overnight. The following day, MDA‐MB‐231 cells at 1 × 10^3^ of each cells/well were added to the BMMs with 10 ng/mL RANKL, treated with different concentrations of nAS‐E, and co‐cultured for 6‐7 days before TRAP staining. TRAP‐positive multinucleated cells with ≧3 nuclei were counted as osteoclasts.

### 2.16 In vivo osteolytic bone metastasis

BALB/cA nude mice (5‐6 weeks old; female) were purchased from Shanghai Sippr‐BK Laboratory Animal Co. Ltd and were maintained at 22‐24°C and 55%‐60% humidity in a room with a 12/12‐hour light/dark cycle. The breast cancer cell line MDA‐MB‐231‐luc was cultured and resuspended in PBS solution to a final concentration of 10^6^ cells/mL. BALB/cA nude mice were inoculated with MDA‐MB‐231‐luc cells directly into the tibiae plateau via a percutaneous approach. The mice were then divided into three groups: the sham group (n = 8), the vehicle group (n = 8) and 20 mg/kg nAS‐E treatment group (n = 8). The mice were treated once a day through intraperitoneal injection for five consecutive days a week, and the treatment lasted for 4 weeks. At the end of the experiment, the metastasis of tumour cells to bones was determined by bioluminescent imaging using an IVIS Lumina XR III in vivo imaging system (Caliper Life Science, USA). Then, Animals were killed and bone density of tibia was assessed with a pQCT (peripheral Quantitative CT, XCT Research SA, Stratec, Germany).

All animal experiments were conducted according to the guidelines of the humane use and care of laboratory animals and were approved by the Shanghai Jiao Tong University School of Medicine Animal Study Committee.

### 2.17 Statistical analysis

All data were expressed as mean ± SD and presented as the mean of triplicate points. One‐way ANOVA and two‐tailed non‐paired Student’s *t* test were used to compare differences, and statistical significance was displayed as *(#) *P* < 0.05 **(##) *P* < 0.01 or ***(###) *P* < 0.001.

## 3 RESULTS

### 3.1 nAS‐E occupied the binding pocket in the KIX‐KID interface identified by molecular docking

**Figure 1:**
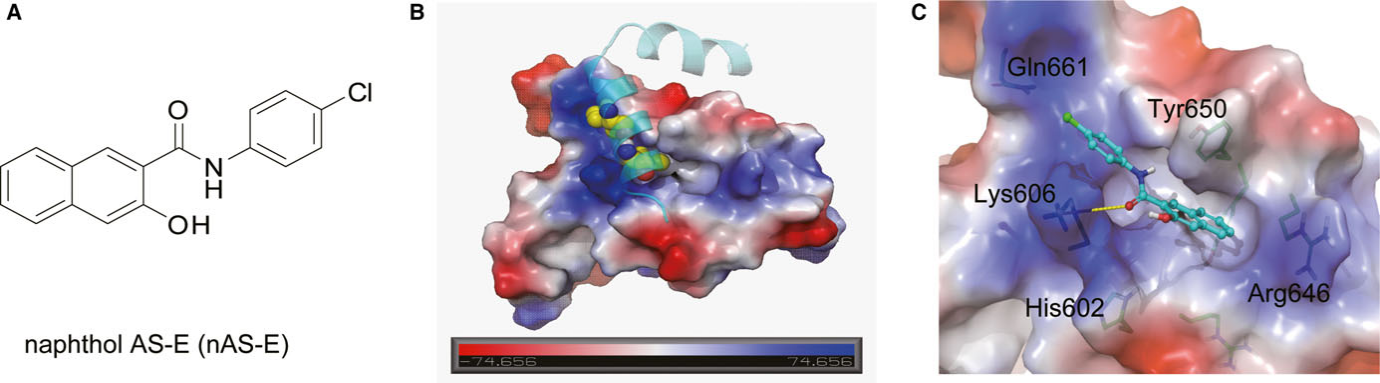
nAS‐E occupied the binding pocket between KIX‐KID interface identified by molecular docking. (A) The chemical structure of nAS‐E. (B) The NMR structures of CREB‐CBP complex downloaded from www.RCSB.org. (C) Detailed interactions between nAS‐E (cyan sticks) and its surrounding residues in KID (blue sticks). The molecular docking simulation against KID binding interface on KIX was performed using Autodock Vina with default settings

The chemical structure of nAS‐E was shown in Figure 1A. In our previous study, we had shown nAS‐E as an inhibitor of the KIX‐KID interaction and CREB‐mediated gene transcription.^47^ To understand the basis of the inhibition of KIX‐KID interaction by nAS‐E, a molecular docking simulation against KID binding interface on KIX was performed to identify the binding mode of nAS‐E (Figure 1B,C). In the final docked structure, the naphthol moiety of nAS‐E was tightly confined within a hydrophobic pocket lined by residues His602, Lys606, Arg646 and Tyr650, and formed T‐shape π‐π interaction with His602 and Tyr650 as shown in Figure 1C. N‐phenylacetamide interacted with Lys606 through cation‐π and hydrogen bond interaction. Electrostatic interaction was observed between chlorine and Gln661.

### 3.2 nAS‐E blocked CREB‐CBP interaction in mouse BMMs

**Figure 2:**
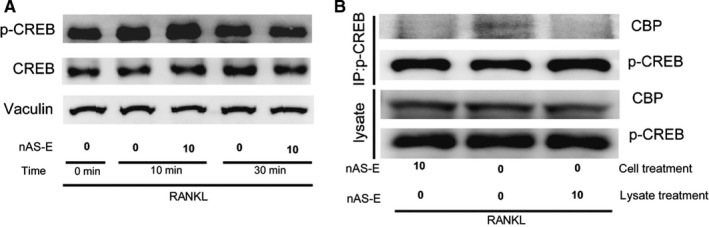
nAS‐E blocked CREB‐CBP interaction in mouse bone marrow monocytes (BMMs). (A) BMMs were pretreated with or without nAS‐E (10 μmol/L) followed by 50 ng/mL RANKL for 0, 10 or 30 min respectively. Then, the expression of p‐CREB and CREB was examined by WB. Vinculin was used as a loading control. (B) The effect of nAS‐E (10 μmol/L) on the interaction of CREB and CBP was examined by co‐IP assays in BMMs, followed by WB analysis

p‐CREB played important roles in CREB signal transduction. To investigate the possibility that p‐CREB might participate in nAS‐E effect on CREB‐mediated gene transcription, we investigated the effect of nAS‐E on p‐CREB levels in BMMs using WB. RANKL, which is the most important cytokine in the differentiation of osteoclasts in BMMs, was incubated with or without nAS‐E in BMMs for 10 and 30 minutes. As shown in Figure 2A, the levels of p‐CREB were up‐regulated by RANKL. However, the treatment of nAS‐E (10 μmol/L) did not decrease p‐CREB, indicating that p‐CREB alteration might not involve in the regulation of nAS‐E on CREB‐mediated gene transcription.

As it is necessary for p‐CREB to recruit the coactivator CBP to help initiate transcription of downstream genes, the effect of nAS‐E on p‐CREB and CBP interaction was thus evaluated by Co‐IP. BMMs were treated with RANKL for 10 min with (lane 1) or without nAS‐E (lane 2 and lane 3). And then, cells were lysed and immunoprecipitated using anti‐p‐CREB antibody, while nAS‐E was added during anti‐p‐CREB immunoprecipitation process (lane 3) (Figure 2B). nAS‐E markedly reduced the levels of bound CBP with p‐CREB as shown in lane 1 and lane 3 compared with lane 2. These results suggested that nAS‐E significantly interrupted p‐CREB and CBP interaction without altering the levels of p‐CREB.

### 3.3 nAS‐E regulated the proliferation, apoptosis and migration of MDA‐MB‐231 breast cancer cells in vitro

**Figure 3:**
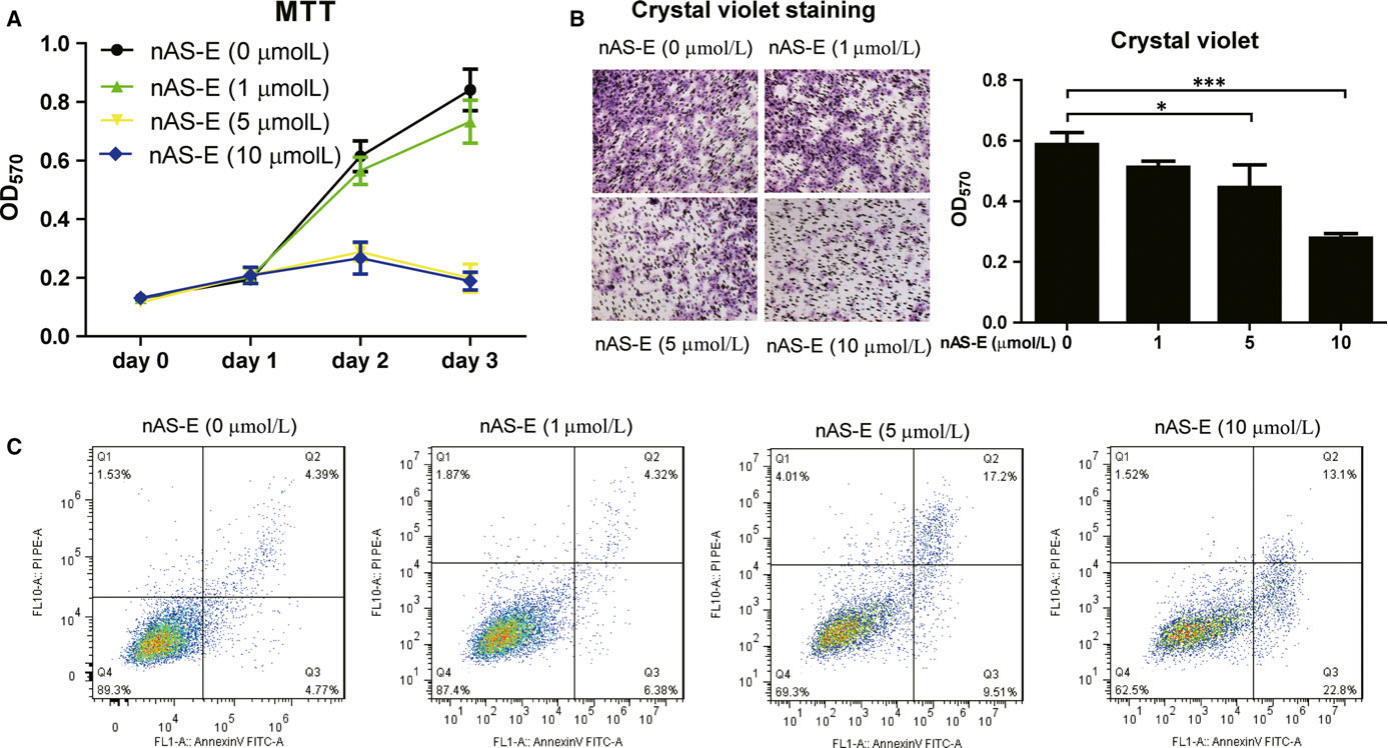
nAS‐E regulated the proliferation, migration and apoptosis of MDA‐MB‐231 breast cancer cells in vitro. (A) Effect of nAS‐E on the proliferation of MDA‐MB‐231 cells. Cells were exposed to different concentrations of nAS‐E as indicated for 1, 2 and 3 days. At the end of each time period, cell viability was measured by MTT assay. (B) nAS‐E inhibited MDA‐MB‐231 cells migration in a transwell assay. Representative images and OD570 indicated the inhibition of migration. Cells were treated for 24 h with nAS‐E (0, 1, 5 and 10 μmol/L). Images magnification: 100×. *P < 0.05 and ***P < 0.001 vs control. (C) nAS‐E induced apoptosis in MDA‐MB‐231 cells. Treated and untreated cells were harvested, washed with PBS, stained with Annexin V‐FITC and PI, and analysed for Annexin V/PI positivity by flow cytometry

To determine the inhibitory effects of nAS‐E on breast cancer cells, the proliferation, migration and apoptosis of MDA‐MB‐231 breast cancer cells were subsequently evaluated following incubation with different concentrations of nAS‐E. In MTT assay (Figure 3A), MDA‐MB‐231 cells proliferation were significantly reduced by 5 and 10 μmol/L nAS‐E from day 2, while 1 μmol/L nAS‐E did not show obvious inhibitory effect on cell proliferation. It is well‐known that cell migration plays a crucial role in tumour metastasis. As shown in migration assay using transwell chambers, an apparent antimobility effect of nAS‐E was illustrated with fewer MDA‐MB‐231 cells compared to the control (Figure 3B). In addition, 5 and 10 μmol/L nAS‐E also increased the apoptosis of MDA‐MB‐231 cells as determined by flow cytometry in Figure 3C.

### 3.4 nAS‐E inhibited RANKL‐induced osteoclastogenesis and bone resorption

**Figure 4:**
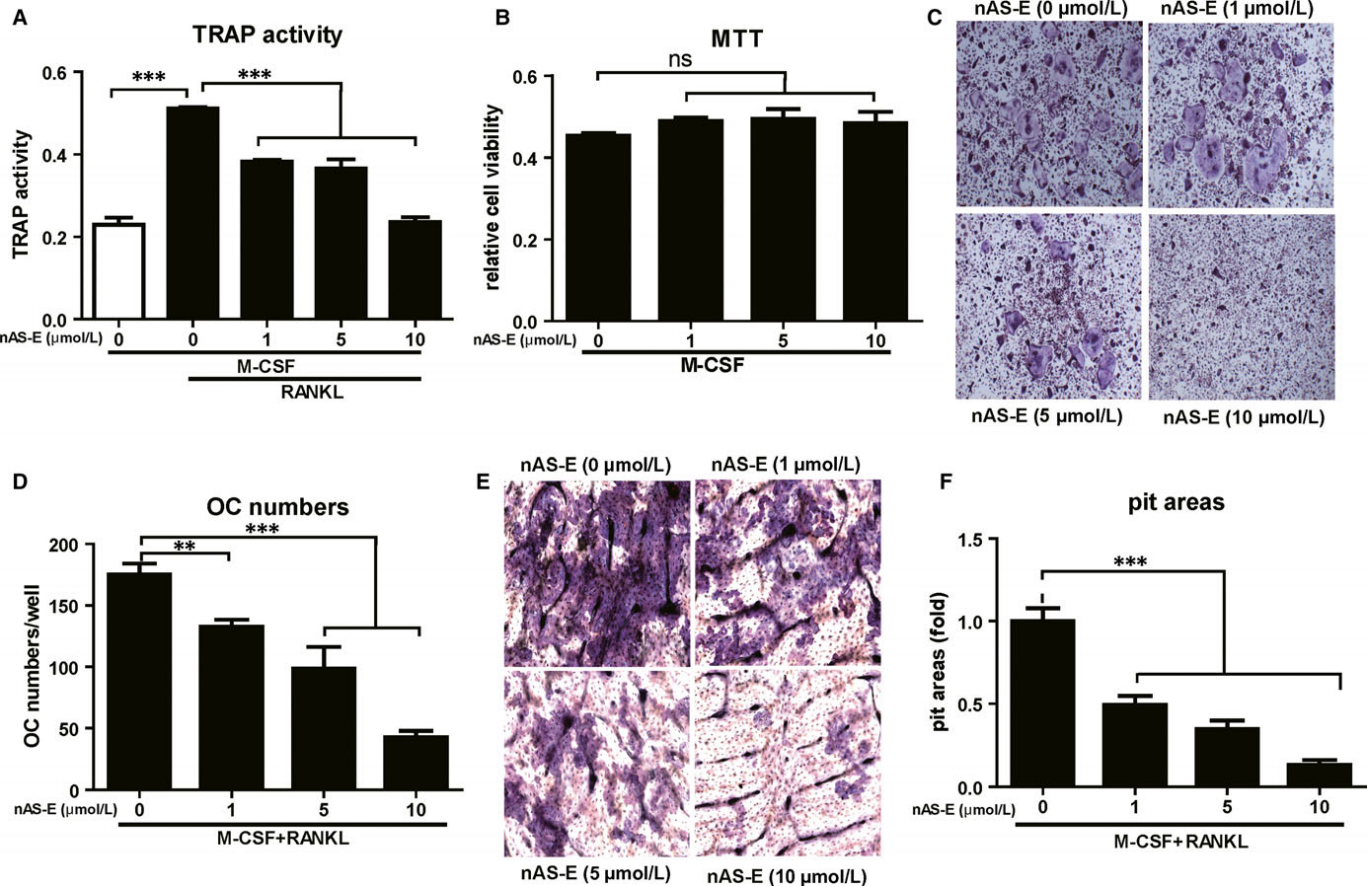
nAS‐E inhibited RANKL‐induced osteoclastogenesis of BMMs and bone resorption. (A) Effect of nAS‐E on TRAP activity induced by 30 ng/mL M‐CSF and 50 ng/mL RANKL. nAS‐E in increasing doses were incubated with BMMs for 2 days in combination with M‐CSF and RANKL. The cell lysates were used to determine the TRAP activity using a TRAP colorimetric assay. ***P < 0.001 vs control. (B) Effect of nAS‐E on BMMs proliferation. Increasing doses of nAS‐E were incubated with BMMs with M‐CSF for 2 days. Cell viability was then determined by MTT assay. “ns” means no significance compared with control. (C) BMMs were treated with various concentrations of nAS‐E, 30 ng/mL M‐CSF and 50 ng/mL RANKL for 4‐6 days and then subjected to TRAP staining. (D) The numbers of osteoclasts from panel C were quantified. **P < 0.01 and ***P < 0.001 vs control. (E) Effect of nAS‐E on bone resorption of osteoclasts. BMMs were plated on bone slices in 96‐well plate and then differentiate into osteoclasts by M‐CSF and RANKL. nAS‐E at indicated concentrations with BMMs for 5‐7 days and then bone slices were fixed and stained by 0.5% toluidine blue to identify bone resorption areas by mature osteoclasts. Images magnification: 200×. (F) The pit areas were quantified using Image J software and presented graphically. The pit area in control was set as 1, these data were expressed as fold change relative to the control. ***P < 0.001 vs control

Tartrate‐resistant acid phosphatase (TRAP) is an established marker for osteoclastogenesis and its activity was highly related with osteoclast differentiation and bone resorption. TRAP activity assay was then conducted to investigate the activity of nAS‐E in BMM osteoclastgenesis induced by M‐CSF and RANKL. As shown in Figure 4A, nAS‐E strongly inhibited TRAP activity in a dose‐dependent manner at the concentrations of 1, 5 and 10 μmol/L without any cytotoxicity as illustrated in MTT assay (Figure 4B). Then, we examined the effects of nAS‐E on the formation of TRAP‐positive multinucleated cells. BMMs were treated with different concentrations of nAS‐E in the presence of M‐CSF and RANKL. As shown in Figure 4C,D, M‐CSF and RANKL induced the formation of TRAP‐positive multinucleated cells, while the treatment of nAS‐E also reduced the formation of osteoclasts dose‐dependently. These results indicated nAS‐E could inhibit osteoclastogenesis of the BMMs induced by M‐CSF and RANKL.

The function of osteoclasts is to disassemble and digest the composite of hydrated protein and mineral, a process known as bone resorption. We thus conducted bone resorption assay to investigate the effects of nAS‐E on osteoclasts activity of bone resorption. Resorption pits formed on the bone slice surface by osteoclasts after stimulation with M‐CSF and RANKL, while a dose‐dependent attenuation of pit areas were observed in the nAS‐E treated groups (Figure 4E). The resorption areas decreased to 48% and 22% after treatment with 5 and 10 μmol/L nAS‐E compared to the control (Figure 4F).

### 3.5 nAS‐E inhibits osteoclast‐specific genes expression and c‐Fos/NFATc1 signalling pathway in vitro

**Figure 5:**
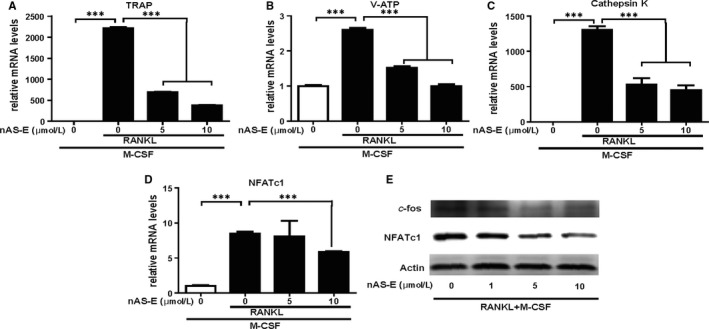
nAS‐E inhibited osteoclast‐specific genes expression and c‐Fos/NFATc1 signaling pathway in vitro. (A‐D) nAS‐E decreased the mRNA expressions of osteoclast marker genes. Osteoclasts marker gene levels of (A) TRAP, (B) V‐ATPase2, (C) Cathepsin K, (D) NFATc1 were determined by quantitative RT‐PCR analysis in BMMs at indicated concentrations of nAS‐E in combination with M‐CSF and RANKL. The results were normalized to β‐actin expression and expressed as fold change relative to gene expression in control cells. ***P < 0.001 vs control. (E) nAS‐E in increasing doses were incubated with BMMs for 3 days in combination with 30 ng/mL M‐CSF and 50 ng/mL RANKL. Then, the expression of NFATc1 and c‐fos was examined by WB. Actin acted as the internal control

RT‐PCR was used to assess the effect of nAS‐E on RANKL‐induced osteoclast‐specific gene expressions during osteoclastogenesis. In the assay, BMMs were treated with M‐CSF and/or RANKL alone or with nAS‐E for 3 days. As shown in Figure 5A‐D, RANKL induced the mRNA expressions of osteogenesis‐related genes such as TRAP, V‐ATPase‐d2 (V‐ATP), Cathepsin K and NFATc1. nAS‐E strongly decreased the expressions of these genes. These results are consistent with decreased osteoclast formation and bone‐resorbing activity. *c*‐fos and NFATc1 are key transcription factors triggering RANKL‐induced osteoclast formation and expressions of related genes. Thus, the protein expressions of c‐fos and NFATc1 were further verified by WB in Figure 5E. These results indicated that nAS‐E, as an inhibitor of CREB‐CBP interaction could effectively down‐regulate the *c*‐fos/NFATc1 signalling in BMMs.

### 3.6 nAS‐E inhibited BMMs osteoclastogenesis induced by MDA‐MB‐231 in vitro

**Figure 6:**
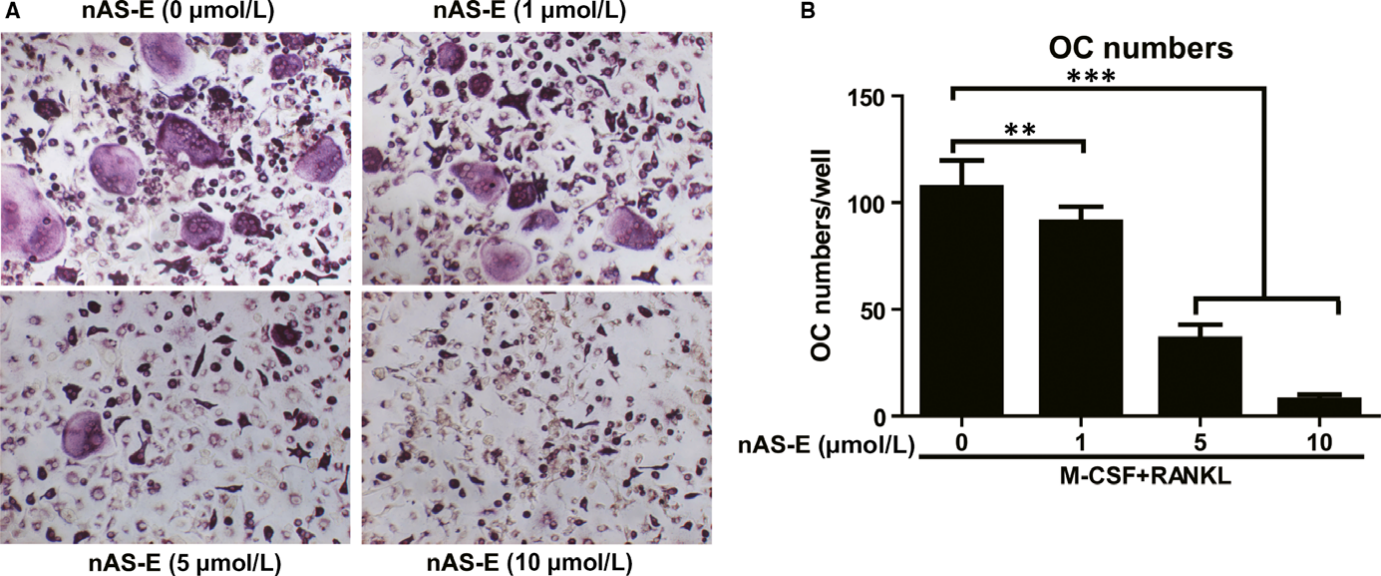
nAS‐E inhibited MDA‐MB‐231 induced BMMs osteoclastogenesis in vitro. (A) BMMs were co‐cultured with MDA‐MB‐231 cells and treated with various concentrations of nAS‐E, as well as 30 ng/mL M‐CSF and 10 ng/mL RANKL for 6‐7 days and then subjected to TRAP staining. (B) The numbers of osteoclasts from panel A were calculated. **P < 0.01 and ***P < 0.001 vs control

Breast cancer bone metastases have a generally osteolytic nature which causes bone loss. We investigated whether nAS‐E could block tumour cell‐induced formation of TRAP‐positive multinucleated cells in BMMs. Figure 6A,B showed that co‐culture of BMMs with human breast cancer MDA‐MB‐231 cells induced osteoclast differentiation and nAS‐E suppressed this effect dose‐dependently. The results suggested that nAS‐E also suppressed the osteoclastogenesis of BMMs induced by tumour cell in vitro.

### 3.7 nAS‐E inhibited breast cancer cell‐induced osteolysis in vivo

**Figure 7:**
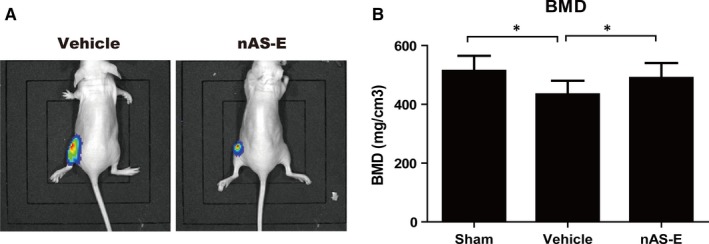
nAS‐E inhibited breast cancer cell‐induced osteolysis in vivo. (A) Representative bioluminescent images from mice subjected to injection of MDA‐MB‐231‐luc cells and treated with vehicle (left) or nAS‐E (right). (B) Quantitative analysis of BMD in tibiae verified that nAS‐E (20 mg/kg) reduced the extent of bone loss induced by MDA‐MB‐231 tumor. *P < 0.05 vs control

Up to now, we have established that nAS‐E had the potential to inhibit osteoclastogenesis and prevent breast cancer‐induced osteoclastgenesis through interrupting CREB‐CBP interaction. Next, we investigated whether nAS‐E could suppress osteolytic bone lesions induced by tumour cells in vivo. The bioluminescent images in Figure 7A showed that nAS‐E treatment retarded the tumour bone metastasis in mice. Quantitative analysis of BMD in tibiae verified that nAS‐E (20 mg/kg) reduced the extent of bone loss induced by MDA‐MB‐231 tumour (Figure 7B). These results suggested that nAS‐E protects against breast cancer‐induced osteolysis.

## 4 DISCUSSION

Breast cancer is one of the most common cancers. Most patients with advanced breast cancer normally develop osteolytic bone metastasis, which are a common cause of morbidity and mortality. There are several agents used to delay the occurrence of bone metastases and reduce the risk of SREs, but they have many limitations and their effects are palliative rather than curative.^18^ Therefore, more effective and safer strategies for treating bone lesions in patients are desperately needed.

CREB was well‐known as a nuclear transcription factor playing a key role in the breast cancer bone metastasis, but strategies targeting CREB signalling pathway such as kinase inhibitors or CREB‐CRE interaction inhibitors might elicit many off‐target effects. However, small organic molecules with good pharmacokinetic properties and specificity, targeting CREB‐CBP interaction to inhibit CREB mediated gene transcription would be better applied as cancer therapeutics.

Our previous study explored small molecule nAS‐E targeting CREB‐CBP interaction as a cell‐permeable inhibitor to inhibit CREB‐mediated gene transcription.^47^ In this study, its effects in breast cancer bone metastasis were further investigated. The binding mode of nAS‐E with KIX‐KID was first confirmed by molecular docking. It was demonstrated that nAS‐E blocked the complex formation of KIX‐KID, the binding interface of CREB‐CBP, via occupying KID binding site through several strong non‐covalent interactions (Figure 1B,C**)**. The binding mode of nAS‐E could be also used to guide further structure optimization. To further verify its mechanism, we used Co‐IP assay to find out whether nAS‐E interrupted p‐CREB and CBP interaction in BMMs. Our results showed that nAS‐E significantly interrupted p‐CREB and CBP interaction without altering the levels of p‐CREB, which indicated that nAS‐E might not exert off‐target effects like the CREB‐related kinase inhibitors.

We also provided evidence that nAS‐E has no cytotoxic effect to BMMs while showed significant inhibition in osteoclast differentiation and ostecoclast function as demonstrated in TRAP activity assay, TRAP staining and bone resorption assay. This result was very important because it suggested that nAS‐E might not have the serious adverse effects from bisphosphonates, which exerted the effects mainly by inducing the apoptosis of osteoclast. Specifically, as shown in Figure S1, nAS‐E showed no cytotoxicity at a concentration of 10 μmol/L on osteoblast cells MC3T3‐E1 and bone marrow mesenchymal stem cells as evaluated by MTT assay, which also indicated that nAS‐E could avoid the adverse effects as a CREB inhibitor. In addition, we also demonstrated that nAS‐E regulated the expressions of nuclear receptor NFATc1 and *c*‐fos that controlled the expressions of bone resorption enzymes such as TRAP, V‐ATP and cathepsin K. We further determined the effects of nAS‐E on the proliferation, migration and apoptosis of MDA‐MB‐231 breast cancer cells by MTT assay, transwell assay and flow cytometric analysis. Therefore, nAS‐E had dual targeting both against MDA‐MB‐231 tumour cells and osteoclasts.

It is reported that breast cancer stimulates RANKL signalling by producing RANKL in the tumour microenvironment. To further evaluate if nAS‐E have the potent to inhibit the cancer cells induced osteoclastogenesis, we also studied its effect in breast cancer‐induced osteoclast genesis in vitro. The results also showed that nAS‐E blocked osteoclastogenesis induced by MDA‐MB‐231 tumour cells.

In addition, the in vivo effect of nAS‐E in protecting against breast cancer‐induced osteolysis was evaluated. The bioluminescent images showed less metastasis signals in nAS‐E treated mice. And the ROI analysis also indicated that nAS‐E could reverse bone loss induced by MDA‐MB‐231 tumour.

AS‐E effectively inhibited osteoclastogenesis and prevented breast cancer‐induced osteoclastgenesis through interrupting CREB‐CBP interaction. Our study provided the first demonstration that small molecules targeting CREB‐CBP interaction represents a novel and promising treatment for the breast cancer‐induced bone loss. It is expected that our results may help for developing novel CREB‐CBP interaction therapeutic agents to treat breast cancer bone metastasis.

## CONFLICT OF INTEREST

The authors declare that they have no conflict of interest.

## AUTHOR CONTRIBUTION

Study design: MJ, XX, XSX, LFD; Study conduct: KY, ZCL, JQ, HBZ, NDQ, QZ, TQW; Data analysis: YFY, MJ, XX; Drafting Manuscript: MJ, XX.
