# Supplementary figures and images for: Small molecule nAS‐E targeting cAMP response element binding protein (CREB) and CREB‐binding protein interaction inhibits breast cancer bone metastasis
Source: J Cell Mol Med. 2018 Nov 20;23(2):1224–34. doi: 10.1111/jcmm.14024 (PMC6349349; doi:10.1111/jcmm.14024)

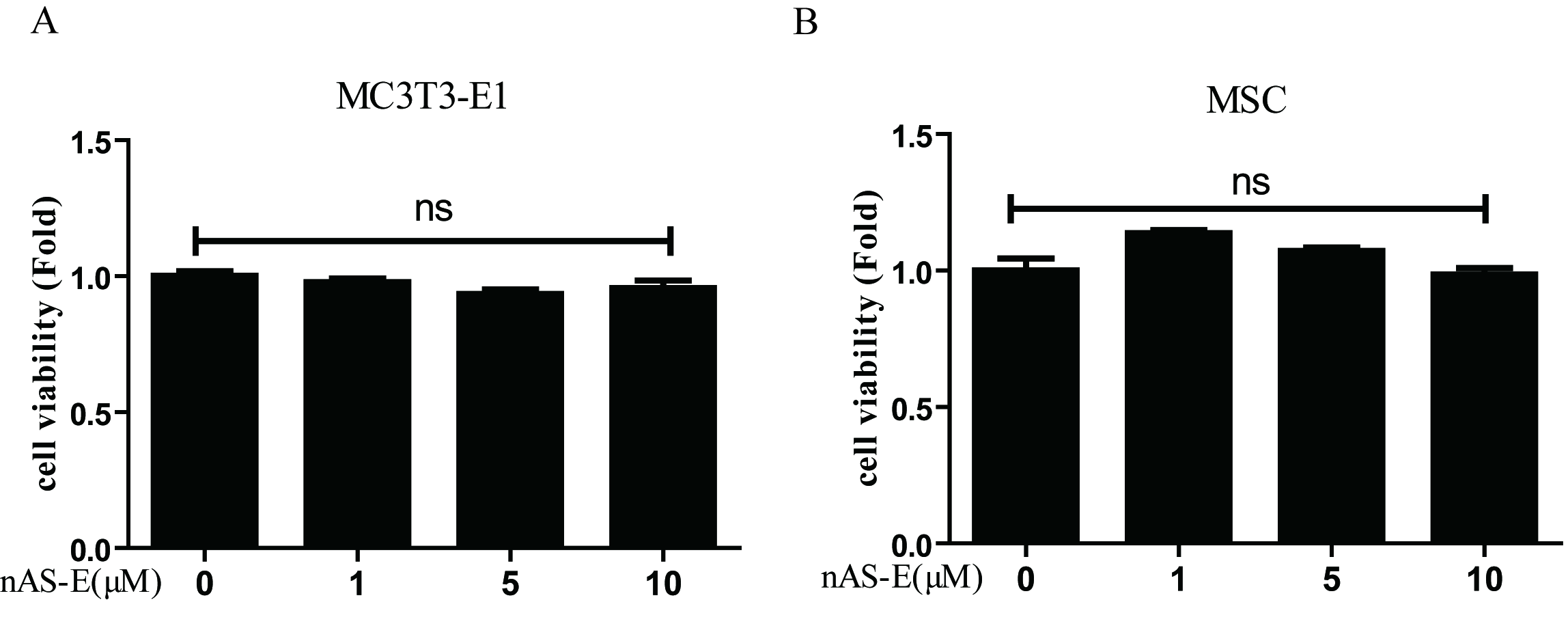

Supplement: Supplementary file 1 [file JCMM-23-1224-s001.tif]

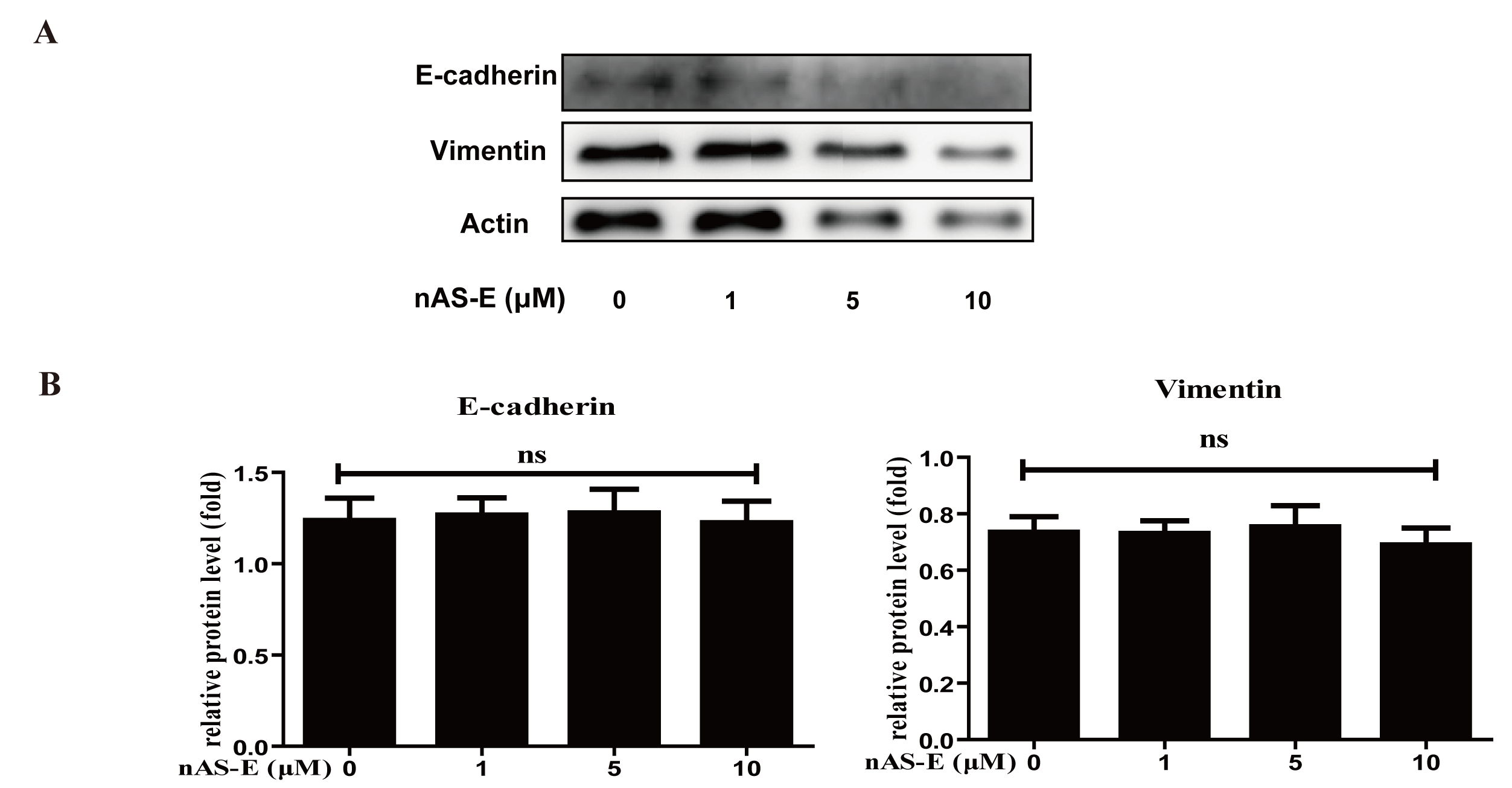

Supplement: Supplementary file 2 [file JCMM-23-1224-s002.tif]
